# Acute Pannexin 1 Blockade Mitigates Early Synaptic Plasticity Defects in a Mouse Model of Alzheimer’s Disease

**DOI:** 10.3389/fncel.2020.00046

**Published:** 2020-03-19

**Authors:** Carolina Flores-Muñoz, Bárbara Gómez, Elena Mery, Paula Mujica, Ivana Gajardo, Claudio Córdova, Daniela Lopez-Espíndola, Claudia Durán-Aniotz, Claudio Hetz, Pablo Muñoz, Arlek M. Gonzalez-Jamett, Álvaro O. Ardiles

**Affiliations:** ^1^Centro de Neurología Traslacional, Facultad de Medicina, Universidad de Valparaíso, Valparaíso, Chile; ^2^Centro Interdisciplinario de Neurociencia de Valparaíso, Universidad de Valparaíso, Valparaíso, Chile; ^3^Programa de Doctorado en Ciencias, Mención Neurociencia, Universidad de Valparaíso, Valparaíso, Chile; ^4^Escuela de Tecnología Médica, Facultad de Medicina, Universidad de Valparaíso, Valparaíso, Chile; ^5^Laboratorio de Estructura y Función Celular, Facultad de Medicina, Universidad de Valparaíso, Valparaíso, Chile; ^6^Centro de Investigaciones Biomédicas, Escuela de Medicina, Universidad de Valparaíso, Valparaíso, Chile; ^7^Center for Social and Cognitive Neuroscience (CSCN), School of Psychology, Universidad Adolfo Ibáñez, Santiago de Chile, Chile; ^8^Biomedical Neuroscience Institute, Faculty of Medicine, University of Chile, Santiago, Chile; ^9^Center for Geroscience, Brain Health and Metabolism, Santiago, Chile; ^10^Program of Cellular and Molecular Biology, Institute of Biomedical Sciences, University of Chile, Santiago, Chile; ^11^Centro Interdisciplinario de Estudios en Salud, Facultad de Medicina, Universidad de Valparaíso, Viña del Mar, Chile

**Keywords:** pannexin 1, Alzheimer’s disease, amyloid-β peptide, synaptic plasticity, p38 mitogen-activated protein kinase (MAPK)

## Abstract

Synaptic loss induced by soluble oligomeric forms of the amyloid β peptide (sAβos) is one of the earliest events in Alzheimer’s disease (AD) and is thought to be the major cause of the cognitive deficits. These abnormalities rely on defects in synaptic plasticity, a series of events manifested as activity-dependent modifications in synaptic structure and function. It has been reported that pannexin 1 (Panx1), a nonselective channel implicated in cell communication and intracellular signaling, modulates the induction of excitatory synaptic plasticity under physiological contexts and contributes to neuronal death under inflammatory conditions. Here, we decided to study the involvement of Panx1 in functional and structural defects observed in excitatory synapses of the amyloid precursor protein (APP)/presenilin 1 (PS1) transgenic (Tg) mice, an animal model of AD. We found an age-dependent increase in the Panx1 expression that correlates with increased Aβ levels in hippocampal tissue from Tg mice. Congruently, we also observed an exacerbated Panx1 activity upon basal conditions and in response to glutamate receptor activation. The acute inhibition of Panx1 activity with the drug probenecid (PBN) did not change neurodegenerative parameters such as amyloid deposition or astrogliosis, but it significantly reduced excitatory synaptic defects in the AD model by normalizing long-term potentiation (LTP) and depression and improving dendritic arborization and spine density in hippocampal neurons of the Tg mice. These results suggest a major contribution of Panx1 in the early mechanisms leading to the synaptopathy in AD. Indeed, PBN induced a reduction in the activation of p38 mitogen-activated protein kinase (MAPK), a kinase widely implicated in the early neurotoxic signaling in AD. Our data strongly suggest that an enhanced expression and activation of Panx1 channels contribute to the Aβ-induced cascades leading to synaptic dysfunction in AD.

## Introduction

Alzheimer’s disease (AD) is an age-dependent neurodegenerative disorder characterized by severe deterioration of cognitive functions leading to dementia (Selkoe, 2001). Classical neuropathological hallmarks of AD, such as intraneuronal accumulation of hyperphosphorylated Tau protein in neurofibrillary tangles or extracellular deposition of amyloid-β peptide (Aβ) in senile plaques, have been considered as the culprit of neuronal damage; however, they show a weak correlation with memory loss (Selkoe, 2001; Nelson et al., 2012). Evidence from human brain studies suggests that the best correlate with cognitive deficits are synaptic loss and hippocampal volume reduction (DeKosky and Scheff, 1990; Terry et al., 1991; Morra et al., 2009; Peng et al., 2015). Accordingly, synaptic dysfunction and memory impairments have been reported in several animal models of AD before the appearance of neuropathological changes (Duyckaerts et al., 2008; Götz and Ittner, 2008; Philipson et al., 2010). In this regard, a number of evidences suggest that soluble oligomeric forms of Aβ (sAβos), identified in AD patients (Gong et al., 2003; Fukumoto et al., 2010) and AD animal models (Mucke et al., 2000; Price et al., 2014), precede the fibrillar amyloid deposition and tau pathology and have been implicated in the synaptopathy observed before the neurodegeneration appearance (Selkoe, 2008; Sheng et al., 2012). Congruently, sAβos from different sources (i.e., synthetic, cell line-derived, human and mouse brain-derived) induce detrimental effects on memory (Cleary et al., 2005; Reed et al., 2011), synaptic morphology (Hsieh et al., 2006; Lacor et al., 2007; Shankar, 2007; Price et al., 2014) and glutamate receptor trafficking, expression and function (Snyder et al., 2005; Hsieh et al., 2006; Shankar, 2007; Miñano-Molina et al., 2011; Ardiles et al., 2012), and consequently, impair excitatory synaptic plasticity (Kim et al., 2001; Townsend et al., 2006; Klyubin et al., 2008; Shankar et al., 2008; Li et al., 2009).

Mechanisms underlying synaptic plasticity manifest as activity-induced long-lasting changes in the synaptic efficacy, which have been better characterized in the CA1 region of the hippocampus (Malenka and Bear, 2004). There, the most prominent forms of excitatory synaptic plasticity are long-term potentiation (LTP) and long-term depression (LTD) of the synaptic strength, which require the activation of N-methyl-D-aspartate receptors (NMDARs) and metabotropic glutamate receptors (mGluRs) for its induction (Malenka and Bear, 2004). Both LTP and LTD are expressed by changes in the trafficking, surface expression, and functionality of glutamate α-amino-3-hydroxy-5-methyl-4-isoxazole propionic acid receptors (AMPARs; Malenka and Bear, 2004) and have been proposed to be the molecular basis of learning and memory (Lynch, 2004; Collingridge et al., 2010; Takeuchi et al., 2014). These modifications in synaptic functionality are accompanied by structural rearrangements in the synaptic connections (Harris et al., 2003; Gogolla et al., 2007; Cingolani and Goda, 2008) manifested as changes in size, shape, and number of dendritic spines (Segal, 2005), which are critically dependent on the remodeling of the actin-cytoskeleton (Matus, 2000; Gordon-Weeks and Fournier, 2014). sAβos have been shown to affect both structural (Hsieh et al., 2006; Lacor et al., 2007; Shankar et al., 2008; Price et al., 2014) and functional synaptic plasticity (Kim et al., 2001; Townsend et al., 2006; Klyubin et al., 2008; Shankar et al., 2008; Li et al., 2009) by inhibiting LTP (Walsh et al., 2002; Wang et al., 2002) and enhancing NMDAR- (Kim et al., 2001; Hsieh et al., 2006; Li et al., 2009) and mGluR5-dependent LTD (Chen et al., 2013; Hu et al., 2014). sAβos-induced LTP inhibition seems to involve excessive activation of extrasynaptic GluN2B-containing NMDARs (Li et al., 2011) and mGluR5s (Li et al., 2009) in a way dependent on the activation of Jun-N terminal kinase (JNK), Cdk5, and p38 mitogen-activated protein kinases (MAPKs; Wang et al., 2004; Li et al., 2011; Rammes et al., 2011) while sAβos-induced LTD requires a metabotropic (non-ionotropic) function of GluN2B-containing NMDAR (Kessels et al., 2013; Tamburri et al., 2013) as well as activation of mGluR5 and their downstream signaling cascades including p38MAPK (Wang et al., 2004; Chen et al., 2013). Although sAβos-induced signaling cascades are relatively well studied, the molecular mechanisms modulating these processes are still poorly understood. Recently, we reported that Panx1, a protein that forms functional nonselective channels in the plasma membrane and that is implicated in cell communication (MacVicar and Thompson, 2010), plays a critical role in modulating the neuronal activity in hippocampal synapses and in controlling the sliding threshold for excitatory synaptic plasticity (Ardiles et al., 2014). This Panx1 function appears to be induced by NMDAR and mGluR5, as glutamate receptors overactivation stimulates ATP release and promote hippocampal hyperactivity, which can be prevented by Panx1 blockers (Thompson et al., 2008; Lopatár et al., 2015). Remarkably, the application of exogenous Aβ in acute hippocampal slices has been shown to induce neuronal death *via* increasing surface membrane expression and activity of Panx1 channels (Orellana et al., 2011b), strongly suggesting the involvement of Panx1 in the Aβ-mediated neurotoxicity. With this in mind, we investigated the participation of Panx1 channels in the synaptic impairments observed in hippocampal tissue of amyloid precursor protein (APP)/presenilin 1 (PS1) transgenic (Tg) mice, a transgenic animal model of AD (Jankowsky et al., 2004). Specifically, we evaluated Panx1 expression and activity in hippocampal tissue of Tg mice and the impact of inhibiting its activity on neurodegeneration parameters, such as Aβ deposition and astrogliosis, and on synaptic plasticity and neuronal structure. Our data show that Panx1 is overexpressed and overactive in Tg hippocampal tissue and that its expression correlates well with enhanced levels of the Aβ peptide. The acute inhibition of Panx1 with probenecid (PBN) reverts the defects in synaptic plasticity and structure observed in hippocampal tissue of Tg mice but has no significant effects on neurodegeneration, suggesting that Panx1 activation plays a major role in the initial steps of the synaptopathy in AD. In fact, PBN significantly reduces the activation of p38MAPK, a kinase that reportedly enhances its expression and activity at early stages of AD (Sun et al., 2003), further supporting a role of Panx1 in the Aβ-induced signaling that leads to the early synaptic dysfunction in AD.

## Materials and Methods

### Animals

Unless otherwise noted, all experiments were carried out in 6-month-old (m.o.) C57BL/6 wild-type (Wt) or APPswe/PSEN1ΔE9 mice (Tg mice). Tg mice, which express the mutant APPSWE (K595N/M596L) and PSEN1ΔE9, deletion of the exon 9 (APP/PS1 mice stock 004462), were obtained from Jackson Laboratory (Bar Harbor, ME, USA). Mice were housed at 22°C at constant humidity (55%), 12/12-h dark-light cycle, with a light phase from 8:00 AM to 8:00 PM. Food and water were provided *ad libitum*. The use and care of the animals were approved by the Ethics and Animal Care Committee of Universidad de Valparaíso (BEA064-2015).

### Drugs and Treatments

PBN and N-[N-(3,5-difluorophenacetyl-l-alanyl)]-S-phenylglycine t-butyl ester (DAPT) were obtained from Sigma Aldrich. SB203580 (SB) was kindly provided by Dr. Andrew Quest (Universidad de Chile). All other chemicals were purchased from Merck or Sigma. Drugs were applied in artificial cerebrospinal fluid (ACSF) as follows: After brain dissection, hemispheres (histology) or slices (electrophysiology and biochemistry) were maintained in ACSF bubbled with a mixture of 5% CO_2_ and 95% O_2_ plus vehicle [sodium hydroxide (NaOH), ethanol, and dimethyl sulfoxide (DMSO)] or drug (100 μM PBN, 1 μM SB, and 0.5 μM DAPT) for 2 h.

### Excitatory Postsynaptic Field Recordings

Hippocampal slices were prepared as we previously reported (Ardiles et al., 2012; Gajardo et al., 2018). Briefly, mice were deeply anesthetized with isoflurane, brains quickly removed and hippocampus sectioned in slices of 350 μm in ice-cold dissection buffer using a vibroslicer (Leica VT1200S, Leica Microsystems, Nussloch, Germany). After 1 h stabilization in ACSF (in mM: 119 NaCl, 26 NaHCO_3_, 1 NaH_2_PO_4_, 11 glucose, 2.5 KCl, 4 CaCl_2_, 4 MgCl_2_, 1.25 NaHPO_4_), slices were treated with 100 μM PBN in ACSF for 2 h and then subjected to stimulation of the Schaffer collaterals using 0.2-ms pulses delivered through concentric bipolar stimulating electrodes and recorded extracellularly in the stratum radiatum of CA1. LTP was induced using four theta burst stimulations (TBSs; 10 trains of four pulses at 100 Hz; 5 Hz inter-burst interval) delivered at 0.1 Hz. LTD was induced using paired-pulse (50-ms interval) low-frequency stimulation (ppLFS; 900 pulses delivered at 1 Hz) in the presence or absence of PBN. LTP and LTD magnitudes were calculated as the average (normalized to baseline) of the responses recorded 50–60 min after conditioning stimulation.

### Synaptosomal Fractionation

Synaptosomes were extracted from hippocampus of 6 m.o. male mice as we previously described (Gajardo et al., 2018). Hippocampi were homogenized in ice-cold homogenization buffer [320 mM sucrose, 4 mM 4-(2-hydroxyethyl)-1-piperazineethanesulfonic acid (HEPES), and 1 mM ethylene glycol-bis(β-aminoethyl ether)-N,N,N′,N′-tetraacetic acid; EGTA), pH 7.4; protease and phosphatase inhibitor cocktails] using a Dounce tissue grinder. The homogenate was centrifuged at 1,000× *g* for 10 min at 4°C (Beckman F0630 rotor) obtaining a supernatant (S1), which was collected, whereas the pellet (P1) was discarded. Then, S1 was centrifuged at 12,000× *g* for 20 min at 4°C. The obtained pellet (P2) containing the membrane proteins was resuspended in homogenization buffer, layered on the top of a discontinuous sucrose density gradient (0.32/1.0/1.2 M) and subjected to ultracentrifugation at 165,000× *g* (Beckman SW-60ti rotor) for 65 min at 4°C. Then, both the sediment and sucrose 0.32/1 M interface were discarded, whereas material accumulated at the interface of 1.0 M and 1.2 M sucrose-containing synaptosome (SP1) fraction was collected. SP1 was diluted with lysis buffer to restore the sucrose concentration to 320 mM and remained on ice with gentle agitation for 30 min. Then, SP1 was centrifuged at 33,000× *g* (Beckman F0630 rotor) for 30 min. The pellet obtained (PS1) was resuspended in a gradient load buffer, loaded on 0.32/1.0/1.2 M discontinuous gradient, and centrifuged at 165,000× *g* (Beckman SW-60ti rotor) for 65 min. The sucrose 1/1.2 M interphase, synaptosome fraction 2 (SP2), was recovered and delipidated in a delipidating buffer. Next, SP2 was diluted with a filling buffer to restore the sucrose concentration and then centrifuged at 33,000× *g* (Beckman F0630 rotor) for 1 h. The sediment obtained (PS2) was washed with 50 mM HEPES-Na and centrifuged at 165,000× *g* (Beckman SW-60ti rotor) for 10 min. The final sediment obtained (PS3), containing postsynaptic densities (PSDs), was resuspended in 50 mM HEPES-Na and homogenized. PS2 or PSD fractions were quantified for protein concentration and submitted to Western blot.

### Western Blot

Hippocampal slices (5–6 slices per animal; seven animals per group) from 3- to 12-months mice were frozen with dry ice and homogenized in lysis buffer [150 mM NaCl, 10 mM Tris-HCl, pH 7.4, ethylenediaminetetraacetic acid (EDTA) 2 mM, 1% Triton X-100, and 0.1% sodium dodecyl sulfate (SDS)], supplemented with a protease and phosphatase inhibitor cocktail (Thermo Fisher Scientific, Rockford, IL, USA) by using a glass-Potter homogenizer. Protein samples from whole homogenates or synaptosomal fractions were centrifuged twice for 10 min at 12,000 rpm at 4°C. Protein concentration was determined with the Qubit^®^ Protein Assay Kit (Thermo Fisher Scientific, Rockford, IL, USA). For both cases, 40 μg of protein per lane were resolved by 10% SDS-polyacrylamide gel electrophoresis (PAGE), followed by immunoblotting on polyvinylidene fluoride (PVDF) membranes (BioRad, Berkeley, CA, USA) and probed with specific antibodies against Panx1 (rabbit anti-Panx1, ABN242 Merck; 1:1,000), PSD-95 (mouse anti-PSD-95, MAB1596 Merck, 1:1,000), synaptophysin (goat anti-SYP, sc-9116, Santa Cruz Biotechnology, Santa Cruz, CA, USA; 1:2,000), p38 MAPK (rabbit anti-p38MAPK, #9212, Cell Signaling Technology, Danvers, MA, USA; 1:1,000), phospho-p38 MAPK (rabbit anti-Thr180/Tyr182, #9211, Cell Signaling Technology, Danvers, MA, USA; 1:1,000), and glyceraldehyde 3-phosphate dehydrogenase (GAPDH; mouse anti-GAPDH, sc-47724, Santa Cruz Biotechnology, Santa Cruz, CA, USA; 1:1,000). Band intensities were visualized by enhanced chemiluminescence kit (ECL, BioRad, Berkeley, CA, USA), and the intensity of each band was scanned and densitometrically quantified using the ImageJ software (version 1.49v; National Institutes of Health, Bethesda, MD, USA).

sAβos were measured by a slot blot assays as previously described (Ardiles et al., 2012). Briefly, the total protein extract was centrifuged at 20,000 *g* for 1 h to eliminate fibrillar aggregates. The protein concentration of the soluble fraction was determined, and 6 mg of protein was spotted in 0.45-mm^2^ nitrocellulose (Millipore, Kankakee, IL, USA), blocked with phosphate-buffered saline with Tween 20 (PBS-T) gelatin 0.4% and incubated using antibodies against total Aβ (6E10, BioLegend; 1:1,000) against oligomeric forms of Aβ (A11; rabbit anti-oligomer, AHB0052, Sigma, 1:5,000) and against Panx1 (rabbit anti-Panx1, ABN242, Merck; 1:1,000). Slot blots were then processed as mentioned above.

### Histology

For histological studies, mice were transcardially perfused with 0.9% NaCl and 4% paraformaldehyde (PFA) in phosphate buffer pH 7.4. For immunohistochemistry, paraffin-embedded brain tissue was cut into 7-μm sections using a cryostat (Leica CM1900) and treated for endogenous peroxidase blockade (3% hydrogen peroxide) followed by CAS-Block (Invitrogen, 008120) and blocking solution (5% goat serum, 0.3% Tween 20 in PBS). Tissues were incubated with primary rabbit anti-Panx1 antibody (ABN242, Merck; 1:200), mouse anti-glial fibrillary acidic protein (GFAP; clone 2E1, Santa Cruz Biotechnology, Santa Cruz, CA, USA; 1:500), and mouse anti-amyloid peptide antibody (clone 6E10, BioLegend; 1:1,000), followed by horseradish peroxidase (HRP)-linked secondary goat anti-mouse or anti-rabbit antibody (Thermo Fisher Scientific, Waltham, MA, USA; 1:250) and diaminobenzidine substrate (Sigma D5637) or p-nitroblue tetrazolium chloride (NBT)/5-bromo-4-chloro-3’-indolyphosphate p-toluidine (BCIP) (Thermo 34042) to visualization of peroxidase or alkaline phosphatase reactions, respectively. Additionally, brain sections were co-stained with Congo red (5%, Sigma) to mark amyloid material or counterstained with Nissl or Kluver–Barrera stains. Images were acquired in an upright microscope (Leica DM500) attached to a Leica ICC50W camera.

For immunofluorescence, 20-μm sections were stained with primary rabbit anti-Panx1 antibody (ABN242, Merck; 1:100), rabbit anti-GFAP (Z0334, DAKO; 1:500), rabbit anti-NeuN (AB177487, Abcam; 1:500), mouse anti-Panx1 antibody (MAB7097, R&D Systems; 1:200), mouse anti-GFAP (clone 2E1, Santa Cruz; 1:250), and mouse anti-amyloid peptide antibody (clone 6E10, BioLegend; 1:500), followed by cyanine-conjugated secondary goat anti-mouse or anti-rabbit antibodies (Jackson ImmunoResearch; 1:500). Additionally, brain sections were co-stained with 4′,6-diamidino-2-phenylindole (DAPI, Sigma; 1:1,000) to label nuclei. Images were acquired in a confocal microscope (upright Eclipse Nikon 80i) using an immersion-oil Plan Fluor 100× magnification objective (NA 1.3) and identical exposure settings between compared samples. The astrocytic burden was measured by estimating the area of GFAP immunostaining regarding the total area (*N* = 5–6 fields from three to four animals per group).

### Ethidium Bromide Uptake Assay

To test the activity of Panx1 channels under basal or glutamate receptor activation conditions, hippocampal slices were stabilized in a chamber with oxygenated ACSF (95% O_2_ and 5% CO_2_) pH 7.4 for 1 h and then incubated with 20 μM of ethidium bromide (EtBr) for 5 min in ACSF only or in the presence of 50 μM of NMDA or 100 μM (S)-3,5-dihydroxyphenylglycine (DHPG). To test the activity of Panx1 and the effect of p38MAPK or γ-secretase inhibitors, hippocampal slices were stabilized for 2 h in the presence of PBN, the p38 MAPK inhibitor SB, or the γ-secretase inhibitor DAPT and their respective vehicles and then incubated with 20 μM of EtBr for 5 min in ACSF. After that, the slices were washed three times during 15 min with ACSF, fixed at room temperature with 4% PFA and 15% sucrose for 30 min, and maintained in PBS buffer. Then, slices were cut into 25-μm sections using a cryostat (Leica CM1900). The sections were stained with primary mouse anti βIII tubulin (clone 5G8, Promega; 1:1,000), rabbit anti-GFAP (Z0334, DAKO; 1:1,000), or mouse anti-NeuN (MAB377, Merck; 1:200), followed by cyanine-conjugated secondary goat anti-mouse or anti-rabbit antibodies (Jackson ImmunoResearch; 1:500). Mounted sections were examined in an Olympus IX81 Custom microscope coupled to an Olympus F-View Monochromatic CCD camera. Images were acquired with a 20× objective using Xcellence Pro software and processed with a custom-made algorithm based on Fiji (ImageJ software). Dye uptake ratio was calculated as the mean fluorescence intensity of the population of positive β-III tubulin or NeuN cells and normalized to the Wt group. At least three fields were selected in every slice.

### Golgi Staining to Dendritic Morphology Visualization

Dissected mouse brains were maintained for 2 h at room temperature in ACSF bubbled with a mixture of 5% CO_2_ and 95% O_2_ in the presence or the absence of 100 μM PBN and then processed for Golgi impregnation following manufacturer’s instructions (FD NeuroTechnologies, Columbia, MD, USA). Coronal sections of 150-μm-thick brain slices were obtained using a semiautomatic cryostat microtome (Kedee KD-2950, Germany) at −20°C and mounted on gelatin-coated slides, developed with solutions of the same kit, dehydrated with a growing battery of alcohols (50–100%), and mounted using Entellan media (Millipore-Sigma, Germany). Images of pyramidal hippocampal neurons were acquired by a Leica Application Suite X (LASX, Leica Microsystems Inc., Buffalo Grove, IL, USA) under bright-field microscopy at 40× or 100× magnification using similar light conditions between experimental groups. Images were digitalized in 1,200 × 1,200 dpi resolution for morphometric analysis.

### Morphometric Analysis

Well-impregnated CA1 pyramidal neurons which had their branching isolated from surrounding neurons and their soma located in the middle third of the tissue were used for analysis. Camera lucida drawings were performed using a 40× objective (Leica M80 attached to Leica DM500). Analysis of the camera lucida drawings were traced using the NeuronJ plugin and then quantified using Sholl analysis macro ImageJ (NIH, Bethesda, MD, USA). Dendritic branching was quantified as the number of intersections with concentric circles at increasing diameters (20-μm steps) placed around the cell body. Dendritic length and spine number were analyzed at 20 μm in Sholl analysis. All morphological analyses were performed blind to the experimental conditions.

#### Statistical Analysis

Data are shown as mean ± standard error of the mean (SEM). Statistical analysis was performed using GraphPad Prism (GraphPad Software Inc., San Diego, CA, USA). Normality distribution of raw data was probed by Shapiro–Wilk test. Unpaired, two-tailed Student’s *T*-tests or Mann–Whitney rank test for two sample comparison, ANOVA followed by Tukey’s or Bonferroni’s *post hoc* test, or Kruskal–Wallis followed by a Dunn’s correction for multiple comparisons were performed to determine significant differences.

## Results

### Pannexin 1 Expression and Activity in Transgenic Mouse Hippocampal Tissue

**Figure 1:**
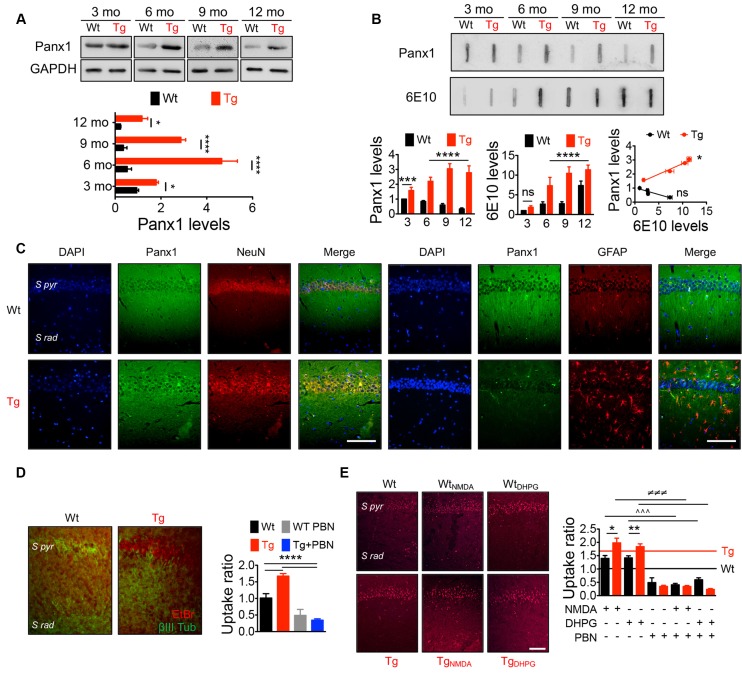
IncreasedPannexin 1 (Panx1) expression and activity in the hippocampus ofAlzheimer’s disease (AD) transgenic (Tg) mice. **(A)**Representative blots of Panx1 expression levels in whole hippocampalhomogenates of wild-type (Wt, black) and Tg (red) mice at 3,6, 9, and 12 months old (m.o.; Top panel). Western blot analysis ofPanx1 levels normalized to glyceraldehyde 3-phosphatedehydrogenase (GAPDH) levels (bottom panel). Two-way ANOVA(*F*_(3,48)_ = 106.3; *p* < 0.0001) for age;(*F*_(1,48)_ = 771.4;*p* < 0.0001) for genotype. **p = 0.0053 for3 m.o.; ****p < 0.0001 for 6 m.o.;****p < 0.0001 for 9 m.o.; ***p = 0.0005 for12 m.o.; (*N* = 7 animals per group) vs. Wt. **(B)**Representative slot blot of Panx1 and total amyloid-β(Aβ) levels immunodetected by 6E10 antibody (6E10 levels) inhippocampal homogenates of Wt and Tg mice at 3, 6, 9, and 12 m.o.(Top panel). Slot blot analysis of Panx1 (left panel) and 6E10(middle panel) levels and correlation between Panx1 and 6E10 levelsat the different ages (right panel). Two-way ANOVA(*F*_(3,48)_ = 11.96; *p* < 0.0001) for age;(*F*_(1,48)_ = 652.4; *p* < 0.0001) for genotype. ****p < 0.0001; *N* = 7 animals per group) forPanx1 levels, followed by Bonferroni’s *post hoc* test(*p* < 0.005). Two-way ANOVA[(*F*_(3,40)_ = 99.60; *p* < 0.0001) for age;(*F*_(1,40)_ = 167.3, *p* < 0.0001) for genotype. **p = 0.0037; ***p = 0.0004; ****p < 0.0001; *N* = 6 animals per group] for levels 6E10, followed by Bonferroni’s *post hoc* test (*p* < 0.005). Two-way ANOVA (*F*_(3,30)_ = 23.43; *p* < 0.0001) for age; (*F*_(1,10)_ = 22.16; *p* = 0.0008). ***p = 0.0004; **p = 0.0013; *p = 0.0260; *N* = 6 animals per group) for 6E10 levels, followed by Bonferroni’s *post hoc* test (*p* < 0.005). Correlation (r^2^ = 0.9725; *p = 0.0138; for Tg; *r*^2^ = 0.8560; ^ns^*p* = 0.0748; for Wt; **C**) Representative images showing the colocalization between Panx1 (green) and NeuN or glial fibrillary acidic protein (GFAP; red) immunoreactivity in the hippocampal CA1 area from 6 m.o. Wt and Tg mice. Scale bar = 50 μm. (**D**) Representative images of ethidium bromide (EtBr) uptake by pyramidal neurons from hippocampal CA1 area treated with 200 μM La^3+^ under resting conditions in the presence or absence of 100 μM of probenecid (PBN; left panel). EtBr uptake ratio normalized to Wt group (right panel). One-way ANOVA (*F*_(3,33)_ = 47.29; *p* < 0.0001) for treatment; ***p < 0.0001 (*N* = 3 animals per group) vs. Wt or Tg, followed by Bonferroni’s *post hoc* test (*p* < 0.005). S pyr, stratum pyramidale; S Rad, stratum radiatum. **(E)** Representative images of EtBr uptake by pyramidal neurons from hippocampal CA1 area treated with 200 μM La^3+^ and stimulated with 50 μM N-methyl-D-aspartate (NMDA) or 100 μM (S)-3,5-dihydroxyphenylglycine (DHPG) in the presence or absence of 100 μM of PBN (left panel). EtBr uptake ratio normalized to Wt group in resting conditions (**D**; right panel). Wt (black line) and Tg (red line) values obtained in resting conditions are indicated. One-way ANOVA (*F*_(11,153)_ = 68.82; *p* < 0.0001) **p = 0.087; (*N* = 3 animals per group) vs. Wt_NMDA_; **p = 0.041, vs. Wt_DHPG_^∧∧∧^*p* < 0.0001, vs. Wt_NMDA_ or Wt_DHPG_; ^≢q≢q≢q^*p* < 0.0001 (*N* = 3 animals per group) vs. Tg_NMDA_ or Tg_DHPG_, followed by Bonferroni’s *post hoc* test (*p* < 0.005). Scale bar = 50 μm.

Panx1 is expressed in several brain areas including neocortex, hippocampus, amygdala, substantia nigra, olfactory bulb, and cerebellum with an age-dependent pattern (Vogt et al., 2005; Ardiles et al., 2014), showing higher levels in embryonic and young tissue, but declining during adulthood (Vogt et al., 2005; Ardiles et al., 2014). To evaluate whether Panx1 expression and distribution were altered in Tg brains, we performed Western blots from whole hippocampal homogenates using tissue from 3- to 12-m.o. Wt and Tg mice (Figure 1A). First, we confirmed that Panx1 expression was reduced during aging in Wt animals. Similarly, Tg also showed an age-dependent reduction, but reaching a peak of expression at 6 m.o. and decreasing after that. However, the expression of Panx1 was greater in whole hippocampal lysates from Tg compared to Wt mice in all the age ranges (Figure 1A). Moreover, a tendency to greater levels of Panx1 was observed in hippocampal synaptosome-enriched fractions isolated from 6-m.o. Tg compared to the Wt brains (Supplementary Figure S1A). Interestingly, the increased Panx1 expression observed in Tg hippocampal tissue significantly correlated with the age-dependent rising levels of Aβ in Tg samples estimated by slot blot experiments using the 6E10 antibody (Figure 1B). Instead, an inverse and nonsignificant correlation between Panx1/Aβ expressions was found in Wt samples (Figure 1B). Similar results were obtained when the anti-oligomer A11 antibody was used to detect Aβ (Supplementary Figure S1B). Since Panx1 has been shown to be expressed not only in pyramidal neurons but also in interneurons and astrocytes (Vogt et al., 2005; Huang et al., 2007), we evaluated whether this increased Panx1expression was given in neuronal or glial cells. As shown in Figure 1C, Panx1 reactivity in hippocampal slices was observed in both NeuN-positive neurons and GFAP-positive astrocytes (Figure 1C). In fact, Panx1-positive astrocytes were significantly more in Tg slices compared to Wt animals (Supplementary Figure S1C). Interestingly, Panx1 exhibited a punctate staining pattern around amyloid plaques, in the pyramidal cell layer, neuropil, and reactive astrocytes (Supplementary Figure S1D), in agreement with previous observations that Panx1 is expressed in reactive astrocytes that are in direct contact with amyloid plaques in this AD model (Yi et al., 2016). These data strongly suggest that Panx1 is overexpressed in the hippocampal tissue of the Tg mice. In order to evaluate whether this augmented Panx1 expression associated with an enhanced Panx1 activity, we performed dye uptake experiments in hippocampal slices from Wt and Tg mice using EtBr in the presence of La^+3^ to block the uptake through connexin hemichannels (Orellana et al., 2011b). As shown in Figure 1D, we observed an increased EtBr fluorescence in hippocampal slices from Tg compared to Wt animals upon basal conditions, which was reduced in the presence of PBN, a Panx1 blocker (Silverman et al., 2008; Figure 1D). Similarly, when we stimulated hippocampal slices with NMDA and DHPG to induce NMDARs and mGluR5 overactivation, we observed a higher EtBr fluorescence in hippocampal slices from Tg compared to Wt tissue (Figure 1E). PBN significantly decreased EtBr fluorescence in both Wt and Tg slices, indicating that dye uptake was through Panx1 channels. Together, these data suggest that, in the AD context, NMDAR and mGluR5 activation induces an exacerbated Panx1 activity.

### Pannexin 1 Blockade With Probenecid Does Not Affect Neurodegeneration-Related Events in Transgenic Mice

**Figure 2:**
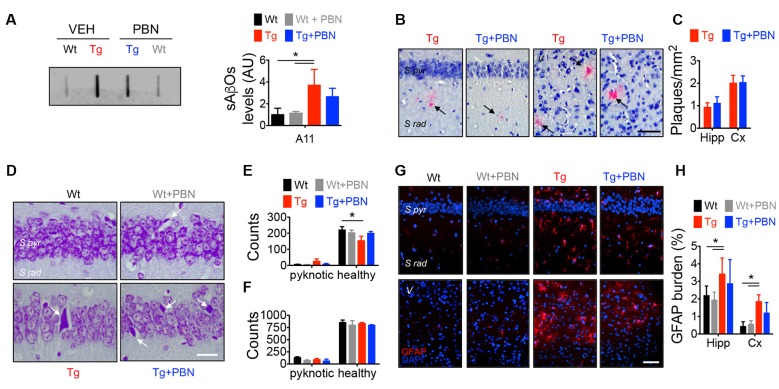
Pannexin 1 (Panx1) blockade does not affect neurodegeneration hallmarks in AD transgenic (Tg) mice. **(A)** Representative slot blot of soluble Aβ oligomers (sAβos) in hippocampal extracts immunodetected by A11 antibody (left panel). Relative levels of sAβos in 6-month-old (m.o.) wild-type (Wt) and Tg mice in the absence (Wt, black; Tg, red) or the presence of 100 μM probenecid (PBN; Wt+PBN, gray; Tg+PBN, blue; right panel). One-way ANOVA (*F*_(3,11)_ = 6.640, *p* = 0.0146; *N* = 3) followed by Tukey’s *post hoc* test (*p* < 0.05). **(B)** Representative images of Kluver–Barrera- and Congo red-stained brain slices from 6 m.o. Tg mice in the absence (Tg, red) or the presence of 100 μM PBN (Tg+PBN, blue). Amyloid plaques are indicated by the arrows. Scale bar = 20 μm. **(C)** Number of amyloid plaques in dendritic layer (S rad.) of CA1 area of hippocampus (Hipp) and layer V (V) of the adjacent cortex (Cx). Unpaired, two-tailed *t-test* did not reveal significant differences (*p* = 0.6070 for number of plaques in Hipp, *p* = 0.9421 for number of plaques in Cx; *N* = 4 per group) vs. Tg. **(D)** Representative images of Nissl-stained brain slices from 6 m.o. Wt and Tg mice in the absence (Wt, black; Tg, red) or the presence of PBN (Wt+PBN, gray; Tg+PBN, blue). Pyknotic cells are indicated by the arrows. **(E,F)** Number of pyknotic and healthy neurons in pyramidal cell layer (S pyr.) of CA1 area of hippocampus (Hipp; **E**) and layer V (V) of the adjacent cortex (Cx; **F**). Two-way ANOVA (*F*_(3,15)_ = 3.910, *p = 0.0302 for healthy cells in hippocampus; *N* = 3 per group) followed by Bonferroni’s *post hoc* test (*p* < 0.05) vs. Wt. **(G)** Representative images of GFAP immunofluorescence in brain slices from 6 m.o. Wt and Tg mice in the absence (Wt, black; Tg, red) or the presence of 100 μM PBN (Wt+PBN, gray; Tg+PBN, blue). **(H)** Astrocytic burden in dendritic layer (S rad.) of CA1 area of hippocampus (Hipp) and layer V (V) of the adjacent cortex (Cx). Scale bar: 200 μm. Two-way ANOVA (*F*_(3,24)_ = 7.115, *p = 0.0014; *N* = 3 per group) followed by Bonferroni’s *post hoc* test (*p* < 0.05) vs. Wt. *S pyr*, stratum pyramidale; S *rad*, stratum radiatum.

Along with amyloid plaques and neurofibrillary tangles, abnormal inflammation including reactive gliosis is one of the neuropathological hallmarks of AD (Selkoe, 2001). In fact, activated astrocytes and microglia surrounding amyloid plaques have been shown in AD brains (McGeer and McGeer, 2003). Accordingly, an increased GFAP staining has been previously reported in this AD model at 6 m.o. (Gomez-Arboledas et al., 2018). Considering that Panx1 channels have been involved in inflammatory conditions (Zhou K. Q. et al., 2019), playing a role in the inflammasome activation in astrocytes and neurons (Silverman et al., 2009), and since PBN appears to exert an anti-inflammatory effect by inhibiting Panx1 activity (Wei et al., 2015; Hainz et al., 2017; Zhang et al., 2019), we evaluated the effect of PBN on sAβos accumulation, amyloid plaques, and GFAP immunoreactivity in Wt and Tg brains (Figure 2). Hippocampal slices were treated during 2 h with 100 μM of PBN and then processed for the evaluation of neurodegeneration parameters. As shown in Figure 2A; sAβos levels, estimated by the A11 antibody, were elevated in whole hippocampal lysates from Tg compared to Wt mice (Figure 2A). Treatment with PBN did not have a significant effect on this Aβ accumulation (Figure 2A). Congo red-positive amyloid plaques were present in the hippocampus and adjacent cortex of Tg mice but were absent in Wt brains (Figure 2B). PBN treatment did not significantly affect the number of amyloid plaques (Figures 2B,C). As previously reported (Gomez-Arboledas et al., 2018), the GFAP-stained area was significantly increased in the hippocampus and adjacent cortex of Tg mice compared to Wt mice (Figures 2G,H and Supplementary Figure S2A), indicative of an augmented astrocytosis. However, treatment with PBN did not affect the percentage of reactive astrocytes (Figure 2H). Similarly, PBN had no effect on the reduced number of neurons in the CA1 pyramidal cell layer and adjacent cortex of Tg mice (Figures 2D,E), indicating that, at least during the 2 h of treatment, there was no impact on neuronal death.

Together, these data indicate that the acute blockade of Panx1 with PBN is not able to interfere with the toxic events associated with the neurodegenerative process in the AD context and suggest that Panx1 overactivation could have a more important role in earlier mechanisms.

### Pannexin 1 Blockade With Probenecid Normalizes Hippocampal Synaptic Plasticity in Transgenic Mice

**Figure 3:**
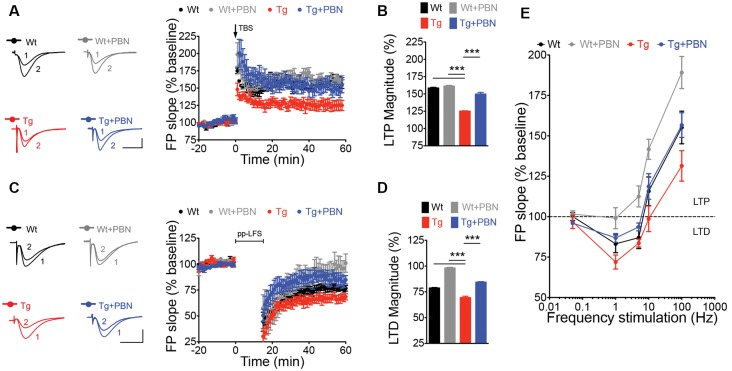
Pannexin 1 (Panx1) blockade reverses synaptic plasticity defects in excitatory hippocampal synapses of AD transgenic (Tg) mice. **(A)** Long-term potentiation (LTP) induced by a theta burst stimulation (TBS) protocol in Schaffer Collateral–CA1 synapse. TBS protocol was delivered at the time indicated by the arrow. Representative traces of field excitatory postsynaptic potentials (fEPSPs) recorded 1 min before (1) and 60 min after (2) TBS. **(B)** Averaged LTP magnitude during the last 10 min of recording for wild-type (Wt) and Tg mice in the absence (Wt, black; Tg, red) or the presence of 100 μM probenecid (PBN; Wt+PBN, gray; Tg+PBN, blue). One-way ANOVA (*F*_(3,39)_ = 143.6, ***p < 0.0001; *N* = 10–17 slices from three to five animals per group) followed by Tukey’s *post hoc* test (*p* < 0.05) vs. Wt. **(C)** Long-term depression (LTD) induced by a paired-pulse low-frequency stimulation (ppLFS) protocol in Schaffer Collateral–CA1 synapse. ppLFS protocol was delivered at the time indicated by the horizontal bar. Representative traces of fEPSPs recorded 1 min before (1) and 60 min after (2) ppLFS. **(D)** Averaged LTD magnitude during the last 10 min of recording for Wt and Tg mice in the absence (Wt, black; Tg, red) or the presence of 100 μM PBN (Wt+PBN, gray; Tg+PBN, blue). One-way ANOVA (*F*_(3,39)_ = 232.1, ***p < 0.0001; *N* = 9–15 slices from three to four animals per group) followed by Tukey’s *post hoc* test (*p* < 0.05) vs. Wt. **(E)** LTP and LTD induced at intermediate frequencies of stimulation (5 and 10 Hz). Calibration: 1 mV, 10 ms. *N* = 8–12 slices from three to four animals per group.

LTP and LTD of the excitatory synaptic strength, the more prominent forms of synaptic plasticity, have been proposed to be the molecular basis of learning and memory (Lynch, 2004; Collingridge et al., 2010; Takeuchi et al., 2014). One of the earliest processes induced by the Aβ signaling in AD is an enhanced glutamate transmission (Palop et al., 2007; Busche et al., 2012) that produces an imbalance in the excitatory synaptic plasticity, impairing LTP, favoring LTD, and leading to synaptic dysfunction (Kim et al., 2001; Walsh et al., 2002; Wang et al., 2002; Hsieh et al., 2006; Li et al., 2009). This latter has been suggested as an initial stage of the disease that correlates well with the early cognitive decline in AD (Forner et al., 2017). Therefore, we analyzed the impact of interfering with Panx1 overactivity on LTP/LTD in acute hippocampal slices of the Tg mice. To do that, we induced synaptic plasticity in hippocampal slices from 6 m.o. mice applying standard theta burst (TBS) and ppLFS protocols to induce LTP and LTD, respectively (Ardiles et al., 2014). Slices were incubated in the presence or absence of 100 μM PBN during 2 h before synaptic plasticity induction. As shown in Figure 3, Tg hippocampal slices exhibited impaired LTP and LTD as compared to Wt slices (Figures 3A,C). The treatment with PBN modified excitatory synaptic responses in Tg slices, reducing LTD and increasing LTP, reaching values comparable to those observed in Wt slices (Figures 3B,D). At intermediate frequencies of stimulation (5 and 10 Hz), PBN produced similar effects, demonstrating that the Panx1 blockade changes the threshold for synaptic modifications in the AD model (Figure 3E). These data support the hypothesis that Panx1 overactivation is involved in the synaptic dysfunction early induced by sAβos.

### Pannexin 1 Blockade With Probenecid Improves Dendritic Morphology and Spine Density in the Hippocampus of Transgenic Mice

**Figure 4:**
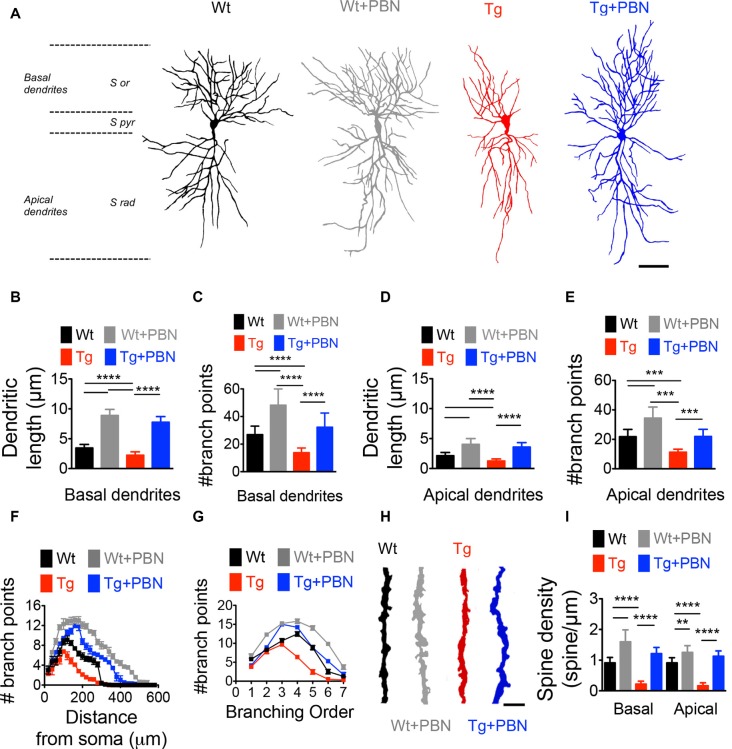
Pannexin 1 (Panx1) blockade reverses the structural defects in dendritic arborization of hippocampal pyramidal neurons of AD transgenic (Tg) mice. **(A)** Representative draws of Golgi impregnated hippocampal neurons from wild-type (Wt) and Tg brains in the absence (Wt, black; Tg, red) or the presence of 100 μM probenecid (PBN; Wt+PBN, gray; Tg+PBN, blue). Scale bar 200 μm. S or, stratum oriens; S pyr, stratum pyramidale; S rad, stratum radiatum. **(B,C)** Quantitative analysis of the length and number of basal dendrites. One-way ANOVA [basal (*F*_(3,240)_ = 169.5; *p* < 0.0001) for basal dendrites; (*F*_(3,240)_ = 983.8; *p* < 0.0001) for basal length; *N* = 61 neurons from three animals per group] followed by Tukey’s *post hoc* test (*p* < 0.05). **(D,E)** Quantitative analysis of the length and number of apical dendrites. One-way ANOVA (*F*_(3,240)_ = 203.8; *p* < 0.0001) for number of apical dendrites; ****p < 0.0001; ***p < 0.001; nonsignificant (n.s.); (*F*_(3,240)_ = 208.5; *p* < 0.0001) for apical dendritic length; *N* = 61 neurons from three animals per group) followed by Tukey’s *post hoc* test (*p* < 0.05). **(F)** Number of the branch points as a function of the distance from soma. Two-way ANOVA (*F*_(3,6,844)_ = 90.076, ***p < 0.0001; *N* = 60 dendrites from three to four animals per group) followed by Bonferroni’s *post hoc* test (*p* < 0.05). **(G)** Distribution of the dendritic arborization number per order. Two-way ANOVA (*F*_(3,1,120)_ = 7.742, ***p < 0.0001; *N* = 61 dendrites from 3 to 4 animals per group) followed by Bonferroni’s *post hoc* test (*p* < 0.05). **(H)** Representative images of dendritic spines in Wt (black), Wt+PBN (gray), Tg (red), and Tg+PBN (blue) groups. Scale bar = 2 μm. **(I)** Quantitative analysis of the spine density. One-way ANOVA (*F*_(3,236)_ = 355.6; *p* < 0.001 for basal dendrites; *F*_(3,236)_ = 492.5; *p* < 0.0001 for apical dendrites; *N* = 60 dendrites from three animals per group) followed by Tukey’s *post hoc* test (*p* < 0.05); ****p < 0.0001; **p = 0.0012.

Activity-dependent modifications in the efficacy of the excitatory synaptic transmission require not only functional changes associated with the trafficking and activity of glutamate receptors but also structural remodeling of the dendritic morphology supporting rearrangements in the synaptic connections (Harris et al., 2003; Gogolla et al., 2007; Cingolani and Goda, 2008). To investigate whether the effect of PBN in the excitatory hippocampal synaptic plasticity in the AD model relies on structural synaptic modifications, we performed Golgi–Cox staining and obtained camera lucida drawings of hippocampal neurons from 6 m.o. of Wt and Tg mice and evaluated dendritic arborization as well as spine density. As shown in Figure 4, Tg pyramidal neurons exhibited a reduction in the dendritic complexity and spine density of CA1 neurons (Figure 4). The total length and the number of branch points of basal and apical dendrites (Figures 4B–E), as well as the dendritic branching throughout the distance from soma (Figure 4F), the branch order (Figure 4G) and the spine density (Figures 4H,I) were significantly lower in Tg hippocampal neurons compared to Wt neurons. Remarkably, PBN antagonized all these effects, suggesting that Panx1 plays a critical role in the early structural synaptic defects observed in the hippocampal tissue of Tg brains. It is noteworthy that PBN also increased dendritic arborization and spine density in hippocampal neurons from Wt animals, further indicating that Panx1 blockade promotes structural changes in neuronal and synaptic morphology.

### The Blockade of Pannexin 1 With Probenecid Reduces p38 Mitogen-Activated Protein Kinase Overactivation in Transgenic Hippocampal Tissue

**Figure 5:**
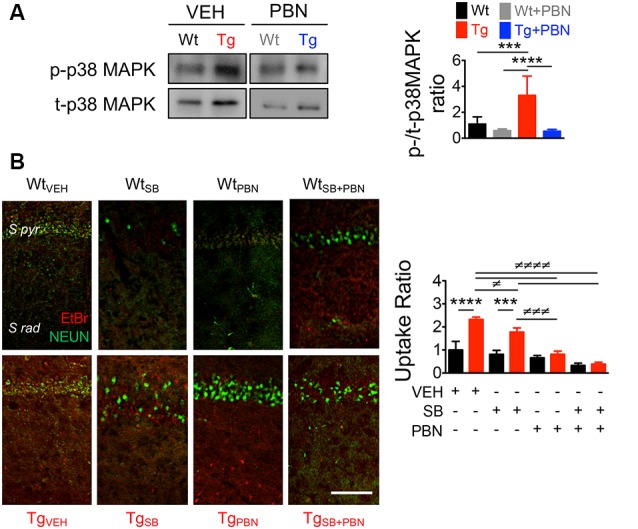
Pannexin 1 (Panx1) blockade reduces p38 mitogen-activated protein kinase (MAPK) activation in hippocampal slices of AD transgenic (Tg) mice. **(A)** Representative blots of p-p38MAPK and t-p38MAPK expression in whole hippocampal homogenates of wild-type (Wt) and Tg mice at 6 months old (m.o.) in the absence (Wt, black; Tg, red) or the presence of 100 μM probenecid (PBN; Wt+PBN, gray; Tg+PBN, blue; left panel). Western blot analysis of phosphorylated p38MAPK levels normalized to total p38MAPK levels (right panel). One-way ANOVA (*F*_(3,20)_ = 15.95; *p* < 0.0001) *N* = 7 animals per group) followed by Bartlett’s *post hoc* test (*p* < 0.05). ***p = 0.0068; ****p < 0.0001. **(B)** Representative images of ethidium bromide (EtBr) uptake by pyramidal neurons from hippocampal CA1 area treated with 200 μM La^3+^ in the presence or absence of vehicle (veh), 50 μM SB203580 (SB), or 100 μM PBN (left panel). EtBr uptake ratio normalized to Wt group with vehicle (right panel). Two-way ANOVA (*F*_(3,16)_ = 63.59; *p* < 0.0001) for treatment; (*F*_(1,16)_ = 74.29, *p* < 0.0001) for genotype. ***p = 0.0002; ****p < 0.0001 (*N* = 3 animals per group) vs. Wt_VEH_; ^≢^*p* = 0.0442; ^≢≢≢^*p* = 0.0002; ^≢≢≢≢^*p* < 0.0001 (*N* = 3 animals per group) vs. Tg_VEH_, followed by Bonferroni’s *post hoc* test (*p* < 0.005).

**Figure 6:**
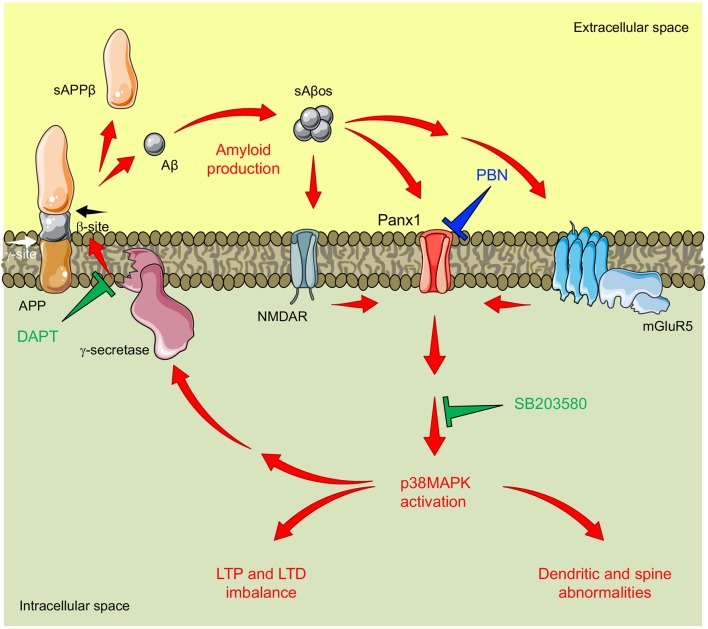
Proposed model for pannexin 1 (Panx1)’s role in AD synaptotoxicity. Amyloid β peptide (Aβ) is generated by the proteolytic cleavage of the amyloid precursor protein (APP). This processing is executed by the consecutive actions of β-secretase (not shown) and γ-secretase resulting in the release of a soluble β-cleaved APP fragment (sAPPβ) and the Aβ, respectively. Aβ can form soluble oligomeric aggregates (sAβos) that easily diffuse and bind to several postsynaptic partners including N-methyl-D-aspartate receptors (NMDARs) and type 1 metabotropic glutamate receptor 5 (mGluR5), enhancing glutamatergic transmission and promoting NMDAR/mGluR5-mediated activation of Panx1 channels. Panx1 overactivity favors signaling cascades that promote the activation (phosphorylation) of p38-MAPK (p38MAPK), a kinase involved in the early stages of the Aβ-induced synaptic dysfunction in AD. In turn, p38MAPK promotes Aβ production and accumulation, further increasing Panx1 overactivity and producing a “positive loop” that amplifies the Aβ-induced neurotoxicity. Congruently with this mechanism, the inhibition of Panx1 with probenecid (PBN) reverses p38MAPK activation and improves LTP/LTD and structural impairments in the AD model brain. Similarly, inhibition of p38MAPK with SB208035 (SB) or the inhibition of γ-secretase-with N-[N-(3,5-difluorophenacetyl-l-alanyl)]-S-phenylglycine t-butyl ester (DAPT) reduces Panx1 overactivity.

A number of evidences demonstrate that the tau-kinase p38MAPK becomes overactive during normal aging as well as in age-related neurodegenerative diseases such as AD (Hensley et al., 1999). Particularly, p38α-MAPK appears to mediate early neuroinflammation, synaptic dysfunction, and spatial memory defects in AD models (Munoz et al., 2007). In fact, p38α-MAPK inhibition reduces the microglial production of pro-inflammatory cytokines, improving synaptic and cognitive functions in Aβ-treated mice (Munoz et al., 2007) and genetic AD models (Roy et al., 2015). In order to analyze whether the role of Panx1 in the synaptic dysfunction in Tg hippocampal tissue relies on the overactivation of p38MAPK, we measured the levels of p38MAPK phosphorylation (p-p38MAPK) in response to the PBN treatment. As shown in Figure 5A, p-p38MAPK was dramatically higher in Tg-total hippocampal lysates compared to the Wt condition. Treatment with PBN significantly reduced p38MAPK phosphorylation, reaching values indistinguishable to those observed in the Wt tissue (Figure 5A). These data strongly suggest that Panx1 activity is an upstream regulator of the p38MAPK signaling in AD. To evaluate whether p38MAPK also influences Panx1 activation, we treated Wt and Tg hippocampal slices with the p38MAPK inhibitor SB, a drug that reportedly reverses Aβ-induced synaptic impairments in mice (Saleshando and O’Connor, 2000; Guo et al., 2017), and measured Panx1-dependent EtBr uptake. As shown in Figure 5B, SB significantly reduced Panx1 activity in Tg slices, suggesting that p38MAPK also favors Panx1 activation in the AD context. These results suggest that a “positive loop” between Panx1 and p38MAPK exacerbates their activities, amplifying Aβ-induced neurotoxicity in AD (Figure 6).

## Discussion

AD is a progressive age-related neurodegenerative condition manifested as a severe deterioration of cognitive functions (Selkoe, 2001). sAβos have been pointed as responsible for the synaptopathy that occurs early in the pathology, causing defects in neuronal morphology (Lacor et al., 2007; Price et al., 2014), alterations in receptor trafficking (Hsieh et al., 2006; Miñano-Molina et al., 2011; Baglietto-Vargas et al., 2018), and consequently impairing excitatory synaptic transmission and plasticity (Sheng et al., 2012). Here, we show that Panx1, a nonselective transmembrane channel that connects intracellular and extracellular spaces (MacVicar and Thompson, 2010), is implicated in the sAβos-induced early synaptic dysfunction observed in a mouse model of AD. In this regard, our data demonstrate that Panx1 is overexpressed in the hippocampal tissue of Tg compared to Wt mice (Figure 1), accumulating in neurons and reactive astrocytes in close proximity to the amyloid plaques (Figure 1C and Supplementary Figure S1), strongly suggesting its association with the Aβ-induced toxicity. Remarkably, the age-dependent overexpression of Panx1 significantly correlates with the Aβ levels in Tg hippocampal tissue (Figure 1B), supporting the idea that Aβ accumulation causes Panx1 overexpression in the AD context. Our results also show an exacerbated Panx1 activity in the hippocampal tissue of the Tg mice upon basal conditions (Figure 1D) and upon the induction of glutamate-transmission with NMDA and DHPG (Figure 1E), which is normalized by the treatment with PBN. Other authors have suggested previously the involvement of Panx1 in the Aβ-induced neurotoxic signaling in the AD neuropathology. Orellana et al. (2011b) reported that the exogenous application of the Aβ peptide in acute hippocampal slices produces neuronal death in a way dependent on glial hemichannel activity and neuronal Panx1 overactivation (Orellana et al., 2011a, b). How does Aβ signaling promote an aberrant function of Panx1 channels? As proposed by Orellana et al. (2011a), Aβ induces microglia activation and the release of factors that potentially promote connexin hemichannel and Panx1 channel opening in astrocytes, causing a nonregulated release of gliotransmitters such as ATP and glutamate, which in turn increases neuronal excitability, triggering neurotoxic cascades (Orellana et al., 2011a, b). Moreover, Panx1 overactivity has also been involved in the Aβ-triggered degranulation of mast cells (Harcha et al., 2015) and in the aberrant gliotransmission that promotes early inflammatory processes in AD mouse models (Yi et al., 2016, 2017), suggesting that neuro-inflammation could directly increase Panx1 expression and activation in the brains of Tg mice. In fact, although it has not been demonstrated in brains, pro-inflammatory stimuli such as interleukin (IL)-1β (Negoro et al., 2013) upregulate Panx1 expression, further supporting this idea. Besides neuro-inflammation, the Aβ peptide can directly impact glutamate receptors, enhancing excitatory synaptic transmission. Aβ signaling has been shown to affect synaptic plasticity by inhibiting LTP (Walsh et al., 2002; Wang et al., 2002) at the expense of enhancing NMDAR- (Kim et al., 2001; Hsieh et al., 2006; Li et al., 2009) and mGluR5-dependent LTD (Chen et al., 2013; Hu et al., 2014). Since Panx1 overactivation has been widely associated with aberrant glutamatergic transmission in several neuropathological conditions (Thompson et al., 2008; Lopatár et al., 2015; Dossi et al., 2018), it is feasible that Aβ upregulates Panx1 activity through a mechanism involving NMDAR and mGluR5 activation. In this regard, it has been demonstrated that Panx1 opening is triggered by the activation of NMDA and mGlu5 glutamate receptors, leading to epileptiform activity in hippocampal neurons (Thompson et al., 2008; Lopatár et al., 2015). More recently, it was shown that Panx1 activity directly contributes to the generation of seizures in human epileptic brain tissues (Dossi et al., 2018). Notably, in all these cases, the pharmacological blockade of Panx1 channels produces an anticonvulsant effect, suggesting that inhibiting Panx1 is an efficient strategy to ameliorate an exacerbated excitatory synaptic transmission. In this regard, the observations that the Panx1 blocker PBN (Silverman et al., 2008) is capable of reversing LTP and LTD impairments in Tg hippocampal slices (Figures 3A–D) support the idea that an exacerbated Panx1 activity could be an important player in the early synaptic dysfunction that affects AD brains. In fact, acute treatment with PBN does not have a significant impact on neurodegeneration parameters such as Aβ deposition, astrogliosis, or neuronal death (Figure 2), further suggesting that Panx1 overactivity becomes more relevant in the initial synaptotoxic mechanisms in AD.

In order to evaluate downstream effectors of the Panx1 overactivity, we estimated the activation of p38MAPK. This tau kinase is overexpressed and becomes overactive during normal aging, as well as in age-related neurodegenerative diseases such as AD (Hensley et al., 1999). Particularly, p38α-MAPK is overexpressed at early stages of AD neuropathology (Sun et al., 2003) and appears to mediate early cascades that lead to neuroinflammation, synaptic dysfunction, and spatial memory defects in AD models (Munoz et al., 2007). Consequently, its inhibition reduces microglial production of pro-inflammatory cytokines and improves synaptic and cognitive functions in Aβ-treated mice (Munoz et al., 2007) and in AD genetic models (Roy et al., 2015). In agreement with the fact that PBN improves LTP/LTD defects in Tg hippocampal slices (Figure 3), our results show that the acute inhibition of Panx1 with PBN significantly reduces p38MAPK activation (Figure 5A), suggesting that Panx1 overactivity contributes to the neurotoxic signaling that leads to p38MAPK activation in AD. In 2017, Colié et al. (2017) demonstrated that the specific neuronal ablation of p38MAPK improves synaptic plasticity and memory performances in an AD model. These authors showed that such effects rely on the reduction of Aβ accumulation (Colié et al., 2017). Interestingly, we do not observe a significant effect of PBN in the Aβ accumulation and deposition (Figures 2A–C), although it has a clear effect on p38MAPK activation (Figure 5A). Perhaps the impact of reducing p38MAPK activity on the Aβ aggregation requires a more long-lasting treatment such as the p38MAPK deletion reported by Colié et al. (2017). Nonetheless, its acute inhibition manages to improve LTP defects in Aβ-treated brain slices (Wang et al., 2004; Origlia et al., 2008; Rutigliano et al., 2018), supporting the idea that drugs that reduce p38MAPK activation, such as PBN (Figure 5A), could be efficient strategies to improve synaptic defects in the AD context. Interestingly, we also observe that the inhibition of p38MAPK with SB, a drug that reportedly reverses Aβ-induced synaptic impairments in mice (Saleshando and O’Connor, 2000; Guo et al., 2017), is capable to reduce Panx1 activity in Tg hippocampal slices (Figure 5B). Since p38MAPK signaling modulates inflammatory cytokine production (Corrêa and Eales, 2012; Colié et al., 2017) and Aβ accumulation (Colié et al., 2017), two factors that increase Panx1 activation and plasma membrane expression (Orellana et al., 2011b; Negoro et al., 2013), it is feasible that a “positive loop” between Panx1 and p38MAPK exacerbates their activities, amplifying Aβ-induced neurotoxicity in AD (Figure 6). Indeed, we observe that the treatment with the anti-Aβ drug DAPT, an inhibitor of the γ-secretase complex (Dovey et al., 2001), efficiently reduces Panx1 overactivation in Tg hippocampal slices (Supplementary Figure S2B), further supporting the idea that p38MAPK-mediated signaling influences Panx1 activity by promoting Aβ accumulation.

Finally, our results show that the inhibition of Panx1 with PBN not only improves synaptic plasticity (Figure 3) but also reverses defects in synaptic structure and dendritic arborization observed in Tg hippocampal tissue (Figure 4). These results are in agreement with our idea that targeting Panx1 overactivity is an efficient strategy to ameliorate early functional and structural synaptic defects in the AD context. Surprisingly, PBN is also able to increase dendritic arborization, bringing the number of branches and dendritic length in Tg hippocampal neurons at levels similar to those exhibited by Wt neurons (Figure 4). Although dramatic, a rapid effect of PBN in neurite and axonal extension has been already demonstrated by other authors (Horton et al., 2017). Since we hypothesize that Panx1 overactivation contributes to the Aβ-induced neurotoxic signaling by favoring p38MAPK activity, it is likely that this tau-kinase be involved in the effect of PBN on dendritic arborization. In fact, p38MAPK activation was recently associated with the mechanisms that induce retardation in the axonal and dendritic outgrowth in offspring mice following maternal neuronal injury (Zhou Y. et al., 2019), supporting the idea that p38MAPK overactivation in the AD context could affect neuronal morphology. As PBN efficiently reduces p38MAPK activation (current Figure 5A), it could impact dendritic arborization in Tg hippocampal neurons for the same reason.

Consistent with our observations in spine density, it was recently shown that Panx1 negatively regulates cortical dendritic spine development and network connectivity (Sanchez-Arias et al., 2019); however, the underlying mechanism has not yet been resolved. Which of the synaptic mechanisms governing the induction of synaptic plasticity could be impacted by an exacerbated Panx1 activity? One possibility is neuronal actin network. Actin is the most prominent cytoskeletal protein at synapses which is expressed in both axonal terminals and dendrites (Cingolani and Goda, 2008). It is highly enriched in dendritic spines where its organization and remodeling support structural modifications that accompany functional changes sustaining synaptic plasticity (Matus, 2000; Tada and Sheng, 2006; Gordon-Weeks and Fournier, 2014). Congruently, neuronal actin cytoskeleton dynamics are importantly perturbed in the AD context (Minamide et al., 2000; Bamburg and Bloom, 2009), producing structural defects that consequently lead to synaptic dysfunctions (Hsieh et al., 2006; Lacor et al., 2007; Shankar, 2007; Sheng et al., 2012). Moreover, it has been reported that Aβ induces aberrant actin polymerization, affecting actin dynamics through a mechanism involving p38MAPK activity (Song et al., 2002). Interestingly, Panx1 channels interact with actin filaments (Bhalla-Gehi et al., 2010) and actin-binding proteins (Wicki-Stordeur and Swayne, 2013; Boyce et al., 2014) and have been shown to participate in the modulation of actin-dependent changes in neuronal morphology (Wicki-Stordeur and Swayne, 2013; Sanchez-Arias et al., 2019). This latter, added to our observations that PBN reverses a deficient dendritic branching and spine density in the Tg hippocampal tissue (Figure 4), strongly suggests that synaptic defects in the AD context rely on Panx1-induced signaling producing actin-dependent structural defects. Further experiments are necessary to confirm this hypothesis.

According to our knowledge, this is the first time that Panx1 is described as a potential target in the early synaptotoxic signaling linked to AD. Furthermore, we provide additional support to the current use of PBN as an emerging tool in clinical and basic research (Colín-González and Santamaría, 2013). PBN is a drug widely used clinically to treat gout and hyperuricemia and also used as a coadjutant to prolong the actions of antibiotic agents (Robbins et al., 2012; Colín-González and Santamaría, 2013). However, it is necessary to note that PBN targets not only Panx1 channels but also other molecules highly expressed at the central nervous system (CNS). Among them, the organic anion transporter 1 (OAT1; Chiba et al., 2011) and the transient receptor potential vanilloid 2 (TRPV2; Bang et al., 2007). Therefore, side effects in brain tissue cannot be ruled out. Nonetheless, PBN has been reported to protect from the excitotoxicity induced by sAβos (Carrillo-Mora et al., 2010) and from the inflammatory conditions in CNS (Wei et al., 2015; Hainz et al., 2017; Zhang et al., 2019). Interestingly, PBN can cross the blood–brain barrier, acting directly on the CNS (Kartzinel et al., 1976; Cowdry et al., 1983) and have been shown to exert neuroprotective effects in several neuropathological contexts including cerebral ischemia–reperfusion (Wei et al., 2015), autoimmune encephalomyelitis (Hainz et al., 2016), sepsis-associated encephalopathy (Zhang et al., 2019), multiple sclerosis (Hainz et al., 2017), and epilepsy (Dossi et al., 2018). Moreover, recent studies reported that gout patients treated with different uricosurics including PBN, exhibit lower risk of developing nonvascular dementia (Hong et al., 2015) and AD (Lu et al., 2016). Although the direct effect of PBN on the cognitive status of AD patients has not yet been evaluated, the fact that in our conditions PBN has positive effects on the early synaptic defects observed in hippocampal tissue of Tg mice paves the way to evaluate in the future its impact on cognitive defects in the AD context.

## Data Availability Statement

The datasets used and analyzed during the current study are available from corresponding authors on a reasonable request.

## Ethics Statement

The use and care of the animals were approved by the Ethics and Animal Care Committee of the Universidad de Valparaíso (BEA064-2015).

## Author Contributions

CF-M performed the experiments and critically revised the manuscript. BG, EM, IG, PMuj, and DL-E performed the experiments. PMuñ, CC, CD-A, CH, and AG-J contributed new reagents/analytic tools and critically revised the manuscript. AG-J wrote the manuscript. ÁA conceived and designed the experiments and wrote the manuscript. All the authors read and approved the final manuscript.

## Conflict of Interest

The authors declare that the research was conducted in the absence of any commercial or financial relationships that could be construed as a potential conflict of interest.
