# Supplementary material for: Acute Pannexin 1 Blockade Mitigates Early Synaptic Plasticity Defects in a Mouse Model of Alzheimer’s Disease
Source: Front Cell Neurosci. 2020 Mar 19;14:46. doi: 10.3389/fncel.2020.00046 (PMC7103637; doi:10.3389/fncel.2020.00046)
Supplement: Supplementary file 1 [file Data_Sheet_1.pdf]

Supplementary Material

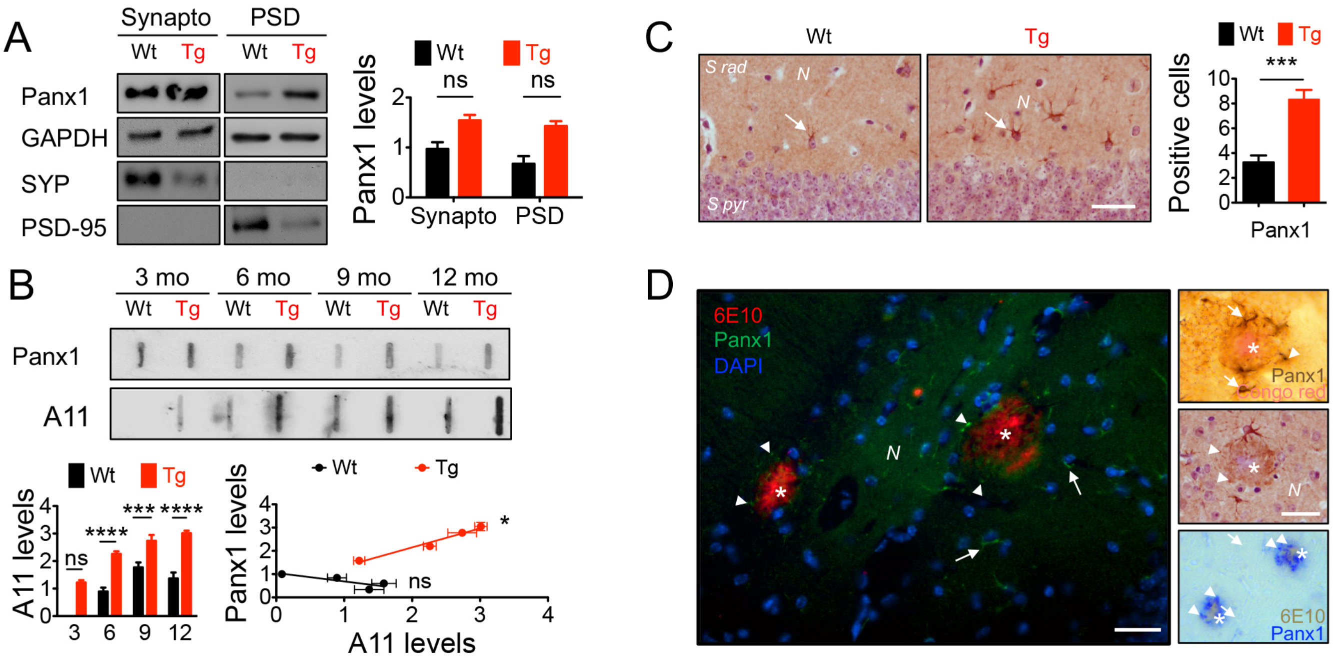

Supplementary Figure 1. Higher levels of Pannexin 1 in the hippocampus of AD transgenic mice. (A) Representative blots of Panx1 expression levels in hippocampal synaptosomes (Synapto)- and postsynaptic densities (PSD)-enriched fractions of 6 months old (mo) wild-type (Wt, black) and transgenic (Tg, red) mice (top panel). Western blot analysis of Panx1 levels normalized to GAPDH (bottom panel). Synaptophysin (SYP) and PSD-95 were included as fraction markers. Mann-Whitney test (^{ns}p=0,1; non-significant for Synapto- and PSD versus Wt; N= 3 animals per group). (B) Representative slot blot of Panx1 and soluble Amyloid-β oligomers levels immunodetected by A11 antibody (A11 levels) in hippocampal homogenates of Wt and Tg mice at 3, 6, 9 and 12 mo (Top panel). Western blot analysis of Panx1 (left panel) and A11 (middle panel) levels and correlation between Panx1 and A11 levels at the different ages (right panel). Two-way ANOVA ([F(3,48)=11,96; p<0,0001] for age; [F(1,48)=652,4; p<0,0001] for genotype. ****p<0,0001; N= 7 animals per group) for Panx1 levels, followed by Bonferroni's post-hoc test (p<0,005). Two-way ANOVA ([F(3,40)=55,41; p<0,0001] for age; [F(1,40)=161,6; p<0,0001] for genotype. ***p= 0,0004; ****p<0,0001; ns: non-significant; N= 6 animals per group) for levels A11, followed by Bonferroni's post-hoc test (p<0,005). Correlation (r^2=0.9714; *p=0.0144; for Tg) (r^2=0.6995; ^{ns}p=0.1636; for Wt)

(C) Representative images of immunohistochemical detection of Panx1 (brown) in the hippocampal CA1 area from 6 mo Wt and Tg mice. Scale bar = 22 µm. Number of astrocytes immunopositive for Panx1. Unpaired two-tailed t-test (***p < 0.0001 for number of positive cells; N=4 per group) versus Wt. (D) Immunodetection of Panx1 in hippocampus from Tg mice by immunofluorescence (left panel) and immunohistochemistry (right panel) exhibiting a puncta distribution (arrow head) around amyloid plaques (asterisk) or present in astrocytes (arrow) and neuropil (N). Scale bar = 20 µm. Amyloid plaques were stained with Congo red, cresyl violet or immunolabeled with the 6E10 antibody. *S pyr*, stratum pyramidale; *S Rad*, stratum radiatum.

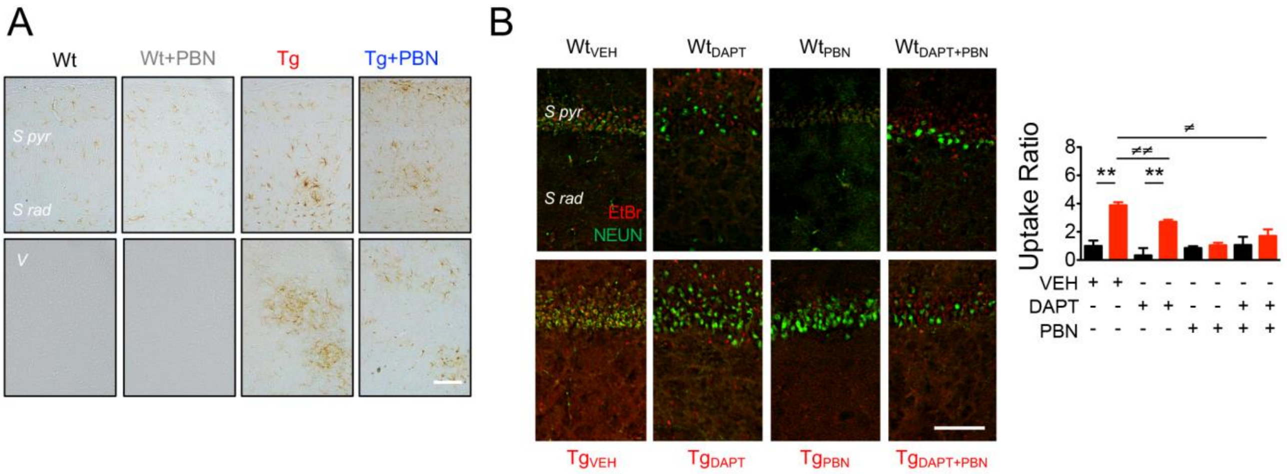

Supplementary Figure 2. Inhibition of γ-secretase reduces Pannexin activity in hippocampal slices of AD transgenic mice. (A) Representative images of GFAP immunoreactivity in brain slices from 6 mo Wt and Tg mice in the absence (Wt, black; Tg, red) or the presence of 100µM PBN (Wt+PBN, gray; Tg+PBN, blue). (B) Representative images of Ethidium bromide (EtBr) uptake by pyramidal neurons from hippocampal CA1 area treated with 200 µM La^{3+} in the presence or absence of vehicle (VEH), 100 µM DAPT or 100 µM PBN (left panel). EtBr uptake ratio normalized to Wt group with vehicle (right panel). Two-way ANOVA ([$F_{(3,16)}$=5.885; p=0,0066] for treatment; [$F_{(1,16)}$=34.94, p<0,0001] for genotype. **p=0,0011; (N= 3 animals per group) versus Wt_{VEH}; **p=0,0081; (N= 3 animals per group) versus Wt_{DAPT} ; ≠p=0,0183; ≠≠p=0,0014; (N= 3 animals per group) versus Tg_{VEH}, followed by Bonferroni's post-hoc test (p<0,005).
